# Impact of the Biocontrol Product, Esquive^®^ WP, on the Indigenous Grapevine Wood Microbiome after a 6-Year Application Period

**DOI:** 10.3390/jof10080566

**Published:** 2024-08-11

**Authors:** Amira Yacoub, David Renault, Rana Haidar, Florian Boulisset, Patricia Letousey, Rémy Guyoneaud, Eleonore Attard, Patrice Rey

**Affiliations:** 1 E2S UPPA, CNRS, IPREM UMR5254, Université de Pau et des Pays de l’Adour, 64000 Pau, France; 2 SAVE, INRAE, Bordeaux Sciences Agro, ISVV, 33882 Villenave d’Ornon, France; 3 Agrauxine by Lesaffre, 49070 Beaucouze, France

**Keywords:** GTDs, Esquive^®^ WP, *Trichoderma atroviride I*-1237, grapevine, microbial communities

## Abstract

Grapevine trunk diseases (GTDs) are currently limiting grapevine productivity in many vineyards worldwide. As no chemical treatments are registered to control GTDs, biocontrol agents are being tested against these diseases. Esquive^®^ WP, based on the fungus *Trichoderma atroviride I*-1237 strain, is the first biocontrol product registered in France to control GTDs. In this study, we determine whether, following grapevine pruning wound treatments with Esquive^®^ WP, changes occurred or not in the indigenous microbial communities that are colonizing grapevine wood. Over a 6-year period, Esquive^®^ WP was applied annually to pruning wounds on three grapevine cultivars located in three different regions. Wood samples were collected at 2 and 10 months after the Esquive^®^ WP treatments. Based on MiSeq high-throughput sequencing analyses, the results showed that specific microbial communities were linked to each ‘region/cultivar’ pairing. In certain cases, a significant modification of alpha diversity indexes and the relative abundance of some microbial taxa were observed between treated and non-treated grapevines 2 months after Esquive^®^ WP treatment. However, these modifications disappeared over time, i.e., 10 months post-treatment. This result clearly showed that Esquive^®^ WP pruning wood treatment did not induce significant changes in the grapevine wood’s microbiome, even after 6 years of recurrent applications on the plants.

## 1. Introduction

Grapevines, one of the most important fruit crops, are of high economic value in many worldwide countries. However, the grapevine is subjected to several microbial diseases that cause huge economic losses [1]. These diseases reduce yields by having a severe impact on the plant’s health but also alter the quality of the wine. To counteract grapevine diseases, growers often rely heavily on chemical products. Nevertheless, the rising use of chemical inputs leads to several negative impacts on environmental and human health. In recent years, the biocontrol of plant pathogens has been intensified in order to reduce pesticide use. The main option for managing plant diseases via biocontrol consists of using antagonistic microorganisms as biocontrol agents (BCAs) of plant pathogens [2,3,4]. Among these beneficial microorganisms, the *Trichoderma* species have emerged as prominent fungi for use as BCAs [5,6,7,8]. Several *Trichoderma* species, such as *T. atroviride*, *T. harzanium*, *T. hamatum*, *T. gamsii*, *T. asperellum*, *T. virens*, etc., have been identified as BCAs against many plant pathogens, and numerous commercial products based on different *Trichoderma* strains are currently available [9,10]. The *Trichoderma* species have several BCA properties that play an important role in the reduction of plant disease incidence and severity. They employ various methods of action that contribute to their beneficial effects. They can counteract the pathogen directly through mycoparasitism, antibiosis, and competition for resources and space or indirectly via the stimulation of plant growth and defense mechanisms [10,11,12,13]. In viticulture, biocontrol has attracted significant interest, especially against pathogens involved in grapevine trunk diseases (GTDs) [14,15,16,17]. The incidence of GTDs has been increasing in the last few years in vineyards worldwide. Consequently, these diseases constitute a major threat to viticulture. They significantly reduce the yield and longevity of the grapevine and thereby degrade the economics of the viticulture industry [18,19].

Esca, botryosphaeria dieback, and eutypa dieback are currently the three main GTDs in worldwide vineyards. Various fungal pathogens are involved in these diseases; *Phaeomoniella chlamydospora*, *Phaeoacremonium minimum*, and *Fomitiporia mediterranea* are the main pathogens involved in esca. The ascomycete *Eutypa lata* is the causal agent of eutypa dieback and more than 26 Botryosphaeriaceae different species have been associated, to date, with grapevine botryosphaeria dieback. In each of these diseases, pathogenic fungi mostly damage wood tissue, causing various types of necrosis and, in the most severe cases, plant death [14,20,21,22,23,24]. Since the ban on sodium arsenate in 2001, in Europe, no effective chemical fungicides have been registered to control GTDs. In that context, research on alternative methods, such as biocontrol using microorganisms, has become essential. BCAs, e.g., bacteria, oomycete, and fungi, are widely used to protect grapevines against the pathogens involved in GTDs [14,15,16,17]. Among the different BCAs used to control GTDs, the *Trichoderma* species were considered the most studied against pathogens involved in these diseases [17]. In France, three commercial products based on the *Trichoderma* species are certified against GTDs: Esquive^®^ WP (the *T. atroviride* I-1237 strain), Vintec^®^ (the *T. atroviride* SC1 strain), and Escalator^®^ (the *T. asperellum* ICC012 strain and *T. gamsii* ICC080 strain). Among these products, Esquive^®^ WP was the first registered product to control pathogens involved in GTDs. According to Mounier et al. [25], applying Esquive^®^ WP for a two-year period on pruning wounds of mature grapevines in the vineyards reduced the expression of foliar symptoms of esca and decreased the rate of plant mortality. Reis et al. [26] showed that pruning wound treatments with Esquive^®^ WP significantly reduced the incidence and severity of two pathogens involved in GTDs, i.e., *Neofusicoccum parvum* and *P. chlamydospora*. Recently, Leal et al. [27] showed that *T. atroviride* I-1237 demonstrated consistent effectiveness against botryosphaeria dieback infections in two different vineyards over three growing seasons. However, other studies pointed out that the Esquive^®^ WP treatment did not reduce infections by the pathogens involved in GTDs [28,29].

In this study, Esquive^®^ WP (*T. atroviride* I-1237) was applied annually and over a 6-year period on the pruning wounds of grapevines from vineyards located in three regions of France. As mentioned above, the efficiency of Esquive^®^ WP against the pathogens involved in GTDs has already been demonstrated. However, the effect of biological control application on the non-targeted wood microbiome is little known. Therefore, our aim here is to assess the biocontrol product’s ability to induce (or not) shifts in grapevine wood’s native bacterial and fungal communities after such a long period of application. The influence of Esquive^®^ WP on the wood necrosis development of treated grapevines was also determined.

## 2. Materials and Methods

### 2.1. Characteristics of the Experimental Vineyards

Samples were collected from four vineyards located in three different French regions. In detail, two vineyards were located in Bourgogne: the first one was planted with cv. Chardonnay grafted on 5BB; it was 24 years old with a density of 7600 vines/ha (Vineyard_1). The second one was planted with cv. Chardonnay grafted on SO4 and was 14 years old with a density of 8500 vines/ha (Vineyard_2). The third vineyard was located in Gironde and was planted with cv. Cabernet Sauvignon grafted on 3309 and was 15 years old (Vineyard_3). This vineyard had a lower density, with 3300 vines/ha, compared to the other studied vineyards. The last vineyard was 16 years old, was situated in Loire-Atlantique, and was planted with cv. Melon de Bourgogne grafted onto an unknown rootstock and had a density of 6500 vines/ha (Vineyard_4). All four vineyards were managed following the common viticulture practices of each region. In each vineyard, the grapevine pruning wounds were treated annually in March by Esquive^®^ WP for 6 years.

### 2.2. Sampling and Experimental Setup

In each vineyard, two samples of 10 asymptomatic plants were carried out 2 months (T1) and 10 months (T2) after receiving pruning wound treatment with Esquive^®^ WP. The pruning wounds were pulverized with the biocontrol product at a dose of 4 kg/ha.

For each vineyard and sampling time point, five out of the ten selected plants were treated with the biocontrol product for a 6-year period, and the five other plants were considered the control. Plants were uprooted and their trunks were transversally cut in order to check the status of the wood. The analyses were performed only on healthy wood, sampled from the inner trunk at two different positions (the trunk head and trunk center) (Figure S1). Overall, 160 wood samples were collected for analysis. All samples were immediately frozen in liquid nitrogen and stored at −80 °C for subsequent analyses.

### 2.3. Necroses Image-Analyses

The trunks of the selected grapevines were cut longitudinally. One half of each trunk was then photographed for the quantification of necrotic wood. The proportion of necrotic wood was measured from the photographs, as described in Khattab et al. [30], by quantitative image analysis using ImageJ freeware software (https://imagej.net/ accessed on 15 Mars 2021) [31]. The relative necrotic area in percentage was calculated by the normalization of the necrotic area to the entire area of the section. In each vineyard, the means of relative necrotic area between treatments were subjected to statistical analyses by parametric tests (ANOVA) followed by pairwise comparisons using Tukey’s post hoc.

### 2.4. Wood DNA Extraction

Wood samples were lyophilized for 24 h and grounded at room temperature using a Tissue LyserII (Qiagen, Hilden, Germany). DNA extraction was performed as described by Pouzelet et al. [32]. Briefly, for each sample, 100 mg of powder was incubated at 65 °C for 1 h in a modified CTAB (hexadecyltrimethylammonium bromide, Sigma-Aldrich, St. Louis, MO, USA) extraction buffer from Doyle and Doyle [33]. The buffer composition was 100 mM Tris–HCl (tris-hydroxymethyl) aminomethane hydrochloride (Sigma-Aldrich, St. Louis, MO, USA); 20 mM EDTA (ethylenediaminetetraacetic acid, Sigma-Aldrich, St. Louis, MO, USA); 1.4 M NaCl; 2% CTAB; 2% PVPP (polyvinylpolypyrrolidone, Sigma-Aldrich, St. Louis, USA); 0.5% *v*/*v* β-mercapto-ethanol (Sigma-Aldrich, St. Louis, MO, USA); 0.4% *v*/*v* of RNase A (Qiagen, Venlo, The Netherlands). After this incubation step, 500 μL of a chloroform/isoamyl alcohol solution (24:1) was added to the solution, mixed, and incubated for 5 min on ice. The mixture was centrifuged for 10 min (2300× *g* at 4 °C). The following steps were performed using a commercial DNeasy plant mini kit (Qiagen, Venlo, The Netherlands) in accordance with the manufacturer’s instructions. All DNA samples were stored at −20 °C.

### 2.5. DNA Sequencing and Data Analysis

After DNA extraction, all samples were shipped in dry ice to the University of Minnesota Genomics Center, UMGC (Minneapolis, MN, USA) for library preparation and sequencing. The primers 515F-806R and ITS1f-ITS2 were used for 16S and ITS amplifications, respectively. All primers were modified to include the Illumina adapters. The full sequences of the different used primers are as follows: ITS1f (CTTGGTCATTTAGAGGAAGTAA) and ITS2 (GCTGCGTTCTTCATCGATGC) for fungal-ITS1 amplification [34], and 515F (GTGYCAGCMGCCGCGGTAA) and 806R (GGACTACN VGGGTWTCTAAT) for bacterial-16S (V4) amplification [35]. DNA sequencing was performed using an in-house Illumina^®^Miseq instrument (Illumina, San Diego, CA, USA) and 2 × 300 paired-end reads.

Fungal and bacterial sequence analyses were bioinformatically treated on a Galaxy server using the FROGS metabarcoding pipeline [36,37] from the portal of the Toulouse Midi-Pyrenees bioinformatics platform. Briefly, sequences were selected according to the following criteria: The bacterial 16S and fungal ITS gene paired-end reads (R1 and R2 reads) were merged with a maximum of 10% mismatches in the overlap region using VSEARCH version 2.22.1 software [38]. Then, reads with no expected length and the ones containing ambiguous bases were discarded. Next, the SWARM clusterization tool was used to cluster the sequences into operational taxonomic units (OTUs) with an aggregation distance of 3 [39]. Chimeric sequences were identified using the tool VSEARCH combined with original cross-sample validation and then removed. Once the chimeric sequences were removed, a final filter was applied to discard the OTUs represented in total by less than five reads [40]. The FROGS ITSx was used to extract the highly variable ITS1 subregion from the ITS sequences; meanwhile, this extraction step was not applied to the 16S sequences. Taxonomy was assigned to the OTUs at a 97% sequence similarity using the Basic Local Alignment Search tool (BLAST version 2.9.0) [41] against the SILVA database, version 123 (https://www.arb-silva.de/contact/ accessed on 14 April 2021) and the UNITE database, version 7.1 (https://unite.ut.ee/cite.php accessed on 14 April 2021) for 16S and ITS, respectively [42]. The OTUs with no kingdom-level classification or matching chloroplast, mitochondrial, or Viridiplantae sequences were then removed from the dataset. Finally, the rarefied OTU table was used as input for the subsequent analyses of the alpha and beta diversities.

### 2.6. Bacterial and Fungal Taxonomy Distribution and Statistical Analysis

Bacterial and fungal community dissimilarities between the individual samples were estimated using Bray–Curtis metrics and the resulting β-diversity was visualized through non-metric multidimensional scaling (NMDS) using MicrobiomeAnalyst [43,44,45]. In order to assess the overall inter-group variance, we performed a non-parametric permutation-based multivariate analysis of variance (PERMANOVA) using the adonis function with a permutation number of 999, available in the vegan package of R version 3.6.2 [46], as implemented in MicrobiomeAnalyst. PERMANOVA was performed to investigate which OTUs significantly differed in abundance among the experimental factors.

The alpha diversity was measured by analyzing the Chao1 richness and Shannon diversity in the Phyloseq package, as implemented in the MicrobiomeAnalyst tool. Species richness and diversity indices (Chao1 and Shannon indexes) were calculated, and the significant differences between treatments were assessed using a one-way analysis of variance (ANOVA) followed by pairwise comparisons using Tukey’s post hoc test (*p* < 0.01).

First, all samples (160) were included in the analysis to evaluate the most discriminant factor for both fungal and bacterial community analyses. Then, in order to focus on the effect of the treatment on the microbe community, analyses were performed in each vineyard separately.

The linear discriminant analysis effect size (LEfSe) algorithm was performed to determine differential abundant taxa in the different vineyards [47]. The online MicrobiomeAnalyst version (2.0) software was used, the threshold for the logarithmic LDA score was set at 1.0, and the Wilcoxon *p*-value was set at 0.01 to identify taxa whose relative abundances were significantly different among vineyards.

The heat tree method was used to compare the abundance at the genus taxonomic level in treated and control plants. The heat tree uses a hierarchical structure of taxonomic classifications to quantitatively (median abundance) and statistically (non-parametric Wilcoxon rank sum test) show the taxon differences among communities. It generates a differential heat tree to show which taxa are more abundant in each treatment. Heat tree analysis was performed using the R metacoder package implemented in MicrobiomeAnalyst. The significant cut-off used was 0.01.

### 2.7. Quantification of T. atroviride Strain I-1237 Using Quantitative PCR Amplification

The qPCR with specific primers for the *T. atroviride* strain I-1237 was carried out on a Stratagene Mx3005P qPCR system (Agilent technologies, Santa Clara, CA, USA). The primers used were as follows: PF74 (5′-GCAGGTCAAAGGCTAAAACG-3′) and PR88 (5′-GCAA GAGGTTGATGGCAGTT-3′) [48]. The single qPCR reactions contained 2 µL of sample DNA, 0.5 µL of each primer at 15 µM and 12.5 µL of 2× SYBR Green Quantitect Master Mix (Qiagen), and H_2_O for 25 µL of the total volume. The qPCR cycling conditions included initial denaturation at 95 °C for 15 min, followed by 40 cycles of denaturation at 95 °C for 15 s, annealing at 60 °C for 30 s, and extension at 72 °C for 30 s; a final extension was performed at 95 °C for 1 min, 60 °C at 30 s, and 95 °C for 30 s. Pure DNA from the *T. atroviride* cultures was used as the corresponding standard. Standard solutions, ranging in concentration from 750 ng/µL to 7.5 × 10^5^ ng/µL, were performed.

## 3. Results

### 3.1. Quantification of Internal Wood Necrosis

**Figure 1:**
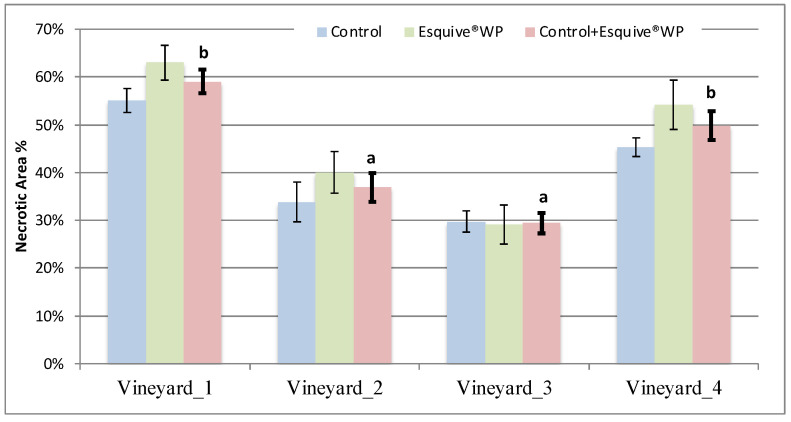
Wood necrosis in plants treated or not with Esquive^®^ WP 10 months after treatment application. The values reported are means (±SE) of 5 samples collected in each treatment. Different letters above bars indicate a significant difference at *p*-value ≤ 0.05 according to Tukey’s post hoc test after ANOVA.

The necrosis area was evaluated 2 and 10 months after the latest Esquive^®^ WP treatment on grapevine plants (Figure S2). Our results showed that irrespective of the studied cultivar, the necrosis areas recorded on Esquive^®^ WP-treated grapevines were not significantly different from those measured in the control plants (Figure 1). However, internal wood necrosis areas were significantly different according to the grapevine’s age and cultivar. For the same cultivar (Chardonnay), wood necrosis observed in 24-year-old plants (Vineyard_1) reached 60% of the total wood, which was significantly higher than the necrosis measured in 14-year-old plants (40%) (Vineyard_2). Moreover, for plants of the same age (Chardonnay (Vineayard_2), Cabernet Sauvignon (Vineayard_3), and Melon de Bourgogne (Vineayard_4), inner wood necroses were significantly different according to the cultivar (Figure 1). The highest level of necrosis area was measured in Melon de Bourgogne plants (50%) compared to Chardonnay and Cabernet Sauvignon plants, with 37% and 30% of the necrosis area, respectively. The same results were obtained for the two sampling time points.

### 3.2. Assessment of T. atroviride Strain I-1237 Wood Colonization

Despite a low limit of detection (2 × 10^−3^ ng/μL), DNA quantities of the *T. atroviride* strain I-1237 have not been detected in our samples.

### 3.3. Sequencing Dataset Description

After paired-end alignments, quality filtering, and the deletion of chimeras and singletons, a total of 5,417,702 bacterial 16S and 1,375,691 fungal ITS1 sequences were generated from 160 samples and assigned to 571 bacterial operational taxonomic units (OTUs) and 257 fungal OTUs.

### 3.4. Analysis of Wood Microbial Community

Overall, the alpha and beta diversities of wood microbiota were mainly affected by the vineyard factor. Beta diversity analysis demonstrated that, among all the studied factors, the vineyard was the primary factor driving the composition of the fungal and bacterial communities, with 23% and 15% of the variance explained, respectively (Table 1). Moreover, significant differences in the measure of alpha diversity indexes (observed and Shannon indexes) were observed in microbial communities according to the vineyard factor, except for the observed index for fungal diversity (Table 2).

Beta diversities of fungal and bacterial communities were also significantly affected according to the tissue factor (trunk head or trunk center) (Table 1). However, the sampling time had a significant effect only on the beta diversity of bacterial communities (*p* < 0.001). Concerning alpha diversity (observed and Shannon indexes), bacterial communities were significantly different according to the tissue and sampling times, whereas for fungal communities, only tissue was a significant factor (Table 2). In regards to the treatment factor, no significant differences were observed in the alpha and beta diversities of fungal and bacterial communities (Table 1 and Table 2).

### 3.5. Vineyard Location/Cultivar Highly Influenced the Fungal and Bacterial Wood Microbiome

**Figure 2:**
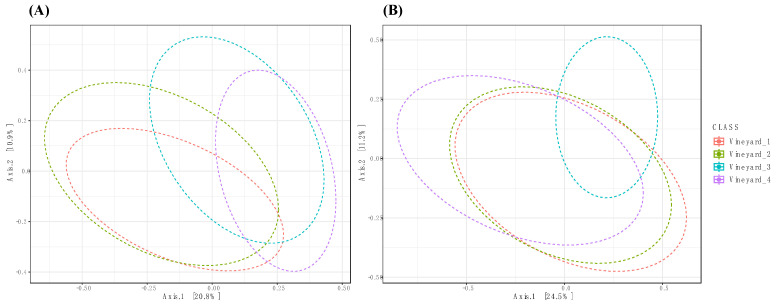
Principal coordinate analysis (PCoA) based on Bray–Curtis dissimilarity metrics, showing the distance of the fungal (**A**) and bacterial (**B**) communities among vineyards.

The principal coordinates analysis (PCoA) of the Bray–Curtis data demonstrated that the vineyard factor was a significant factor in explaining the differences in fungal and bacterial community diversities (Table 1 and Figure 2). PERMANOVA pairwise comparisons showed that fungal communities were significantly different (*p* = 0.001) between all vineyards. Regarding bacterial communities, the same result was obtained (*p* = 0.001), except for Vineyard 1 vs. Vineyard 2. Indeed, wood bacterial communities were not significantly different in the two Chardonnay vineyards (*p* = 0.07) (Table S1).

**Figure 3:**
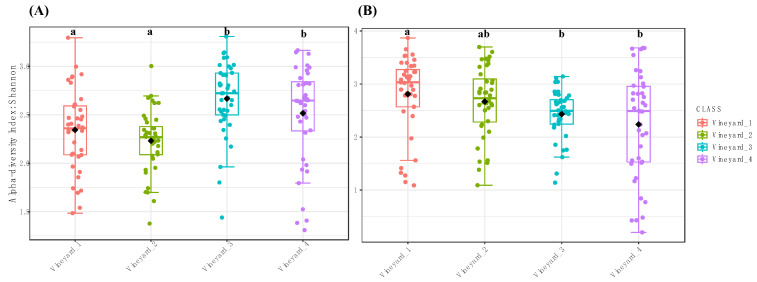
Boxplot illustrating the differences in Shannon diversity measures of the fungal (**A**) and bacterial (**B**) communities between vineyards. Different letters above bars indicate a significant difference at *p*-value ≤ 0.01 according to Tukey’s post hoc test after ANOVA.

Significant differences in the measures of alpha diversity (observed and Shannon indexes) at the genus level were found between the bacterial and fungal wood microbiome in the different vineyards (Figure 3 and Figure S3). Shannon’s estimator indicated that fungal diversity in both Chardonnay vineyards (Vineyard_1 = Shannon: 2.35 and Vineyard_2 = Shannon: 2.25) was significantly lower than those observed in Cabernet Sauvignon (Vineyard_3 = Shannon: 2.75) and Melon de Bourgogne (Vineyard_4 = Shannon: 2.65) cultivars (Figure 3A). The pairwise comparison (post hoc) showed that there are no significant differences between the fungal communities of Vineyard_1 and Vineyard_2 (*p*-value = 0.3). Moreover, the Vineyard_3 and Vineyard_4 fungal communities are similar (*p*-value = 0.15) (Figure 2A). The richness index (observed) indicated that there were no significant differences in fungal communities between the different vineyards (Figure S3A).

Regarding bacterial communities, the Shannon and observed indexes indicated that bacterial richness and diversity are significantly different between vineyards (Figure 3B and Figure S3B). The Shannon index, measured in Vineyard_1 (Shannon: 2.8), was significantly higher than those measured in Vineyard_3 (Shannon: 2.5) and Vineyard_4 (Shannon: 2.25). However, the pairwise comparison (post hoc) of the Shannon index measures showed that the bacterial microbiome of Chardonnay vineyards (Vineyard_1 and Vineyard_2) was similar (*p*-value *=* 0.37). Additionally, no significant differences were observed in bacterial communities of the young vineyards (Vineyard_2, Vineyard_3, and Vineyard_4) (*I* < 0.01). Observed index analyses showed that only bacterial communities in Vineyard_3 were significantly different from those observed in all the other studied vineyards (*p*-value < 0.01) (Figure S3B).

**Figure 4:**
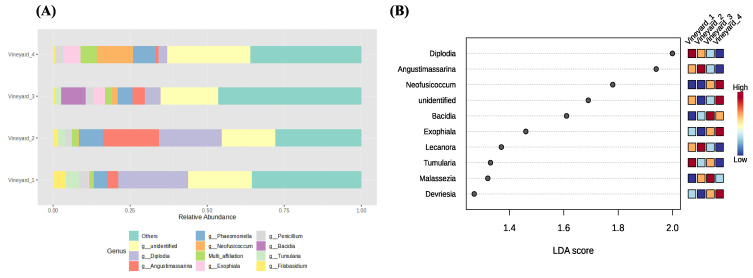
Wood fungal community relative abundance in different vineyards. (**A**) shows the most differentially abundant taxa between vineyards. (**B**) LEfSe analysis shows the genera with significant differential abundances in the different vineyards. The colors in the heatmap represent the abundances of genera.

**Figure 5:**
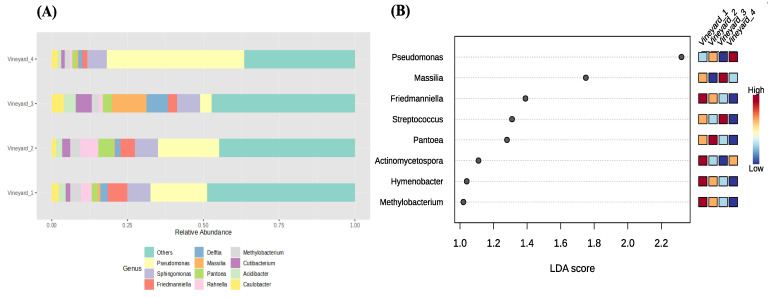
Wood bacterial community relative abundance in different vineyards. (**A**) shows the most differentially abundant taxa between vineyards. (**B**) LEfSe analysis shows the genera with significant differential abundances in the different vineyards. The colors in the heatmap represent the abundances of genera.

The relative abundance of the main genera detected in the different vineyards is shown in Figure 4A and Figure 5A). The bar plots in Figure 4A provide an overview of the key differences in fungal taxa abundance depending on the vineyard. More precisely, we showed that the most abundant fungal taxa were different according to the vineyards (Figure 4A). *Diplodia* was the most abundant taxa in Vineyard_1 with 23% of relative abundance. However, in Vineyard_2, the *Diplodia* and *Angustamassarina* taxa were the most abundant, with about 20% and 18% of relative abundance, respectively. Regarding Vineyard_4 and Vineyard_3, *Neofusicoccum* (12% of relative abundance) and *Bacidia* (8% of relative abundance) were the most abundant genera, respectively. *Neofusiccocum* taxa were 100 and 10 times more abundant in Vineyard_4 and Vineyard_3, respectively, than in both Chardonnay vineyards (Vineyard_1 and Vineyard_2). *Phaeomoniella* was among the three most abundant taxa in all vineyards. Surprisingly, the relative abundance of the *Trichoderma* genus was very low (<0.1%) in all vineyards.

LEfSe analysis detected that 22 taxa explained the differences in the analyzed fungal communities among vineyards. *Diplodia* (LDA score 2) and *Angustimassarina* (LDA score 1.95) were the major genera that contributed to the differentiating wood fungal communities associated with vineyards, being most abundant in the wood samples collected in Vineyard_1 and Vineyard_2. The *Neofusicoccum* (LDA score 1.75) genus also determined the dissimilarities among vineyards; however, this genus was more abundant in Vineyard_4 and Vineyard_3 than in Vineyard_1 and Vineyard_2.

Regarding the relative abundance of bacterial communities, the bar plots in Figure 5A provide a detailed analysis of their composition according to the vineyards. As for the fungal communities, the results showed that the most abundant bacterial species were different according to the vineyards (Figure 5A). *Pseudomonas* was the most abundant taxa in Vineyard_1, Vineyard_2, and Vineyard_4, with 18%, 20%, and 45% of relative abundance, respectively. However, the *Massilia* genus was the most abundant one in Vineyard_3, with 12% of relative abundance. The *Sphingomonas* taxa were the second-most abundant species in all vineyards, with about 7% of relative abundance. The *Pantoea* and *Rahnella* taxa were more abundant in Vineyard_2, with 5% relative abundance than in the other vineyards.

The linear discriminant analysis effect size (LEfSe) detected that nine genera determined the dissimilarities between vineyards—the more prevalent genera after the LEfSe analyses are represented in Figure 5B. *Pseudomonas*, enriched in Vineyard_4, was the major genus that contributed to the differentiating wood bacterial communities associated with vineyards. The *Massilia* genus also determined the dissimilarities among vineyards appearing more abundant in Vineyard_3.

### 3.6. Effect of Esquive^®^ WP Treatment on Wood Microbial Community Composition

Given that the vineyard factor is the primary source of structural difference in the microbial communities, the subsequent analyses were performed in each vineyard separately and at two different sampling times (2 and 10 months after Esquive^®^ WP treatment) and in two different wood tissues (the trunk head and trunk center).

#### 3.6.1. Esquive^®^ WP Affects Temporary Wood Microbial Diversity Two Months Post Treatments

**Figure 6:**
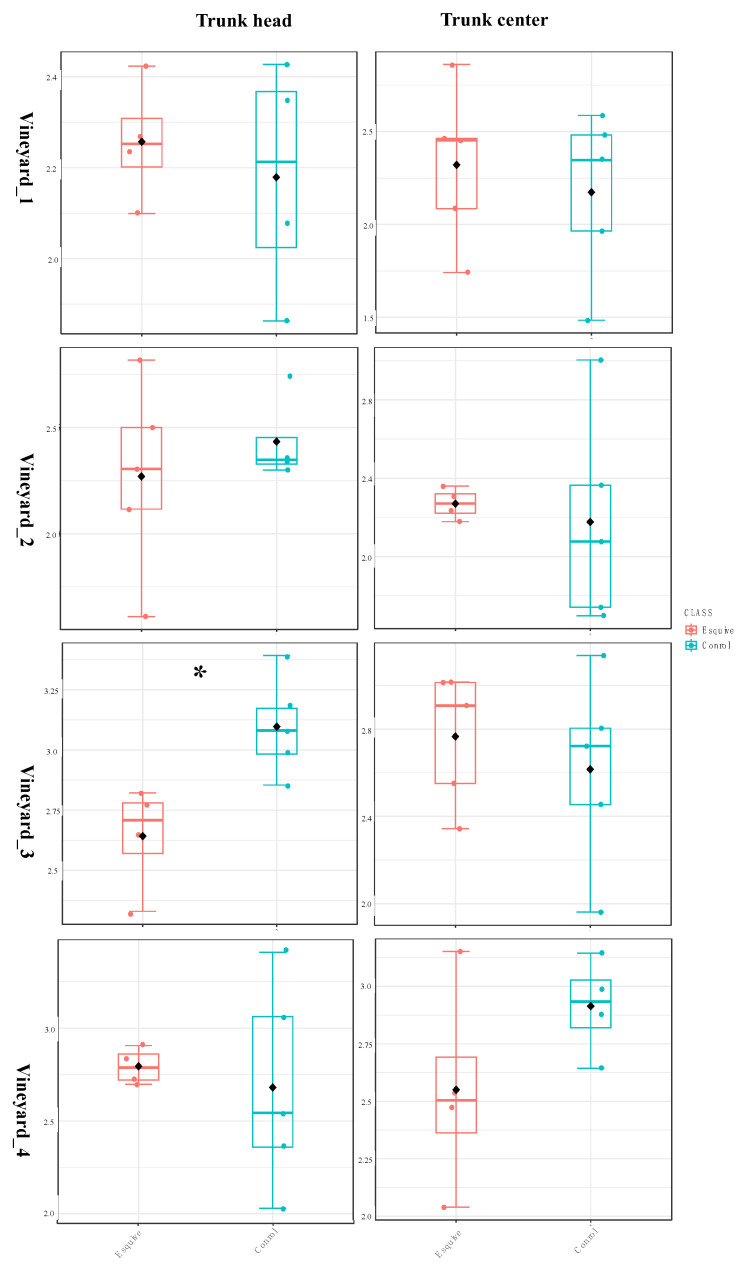
Boxplot illustrating the differences in Shannon diversity measures of the fungal communities in the grapevine healthy wood of Esquive^®^ WP-treated and control plants at 2 months post-inoculation (T1). * Indicates significant differences between treatments (*p* < 0.01).

**Figure 7:**
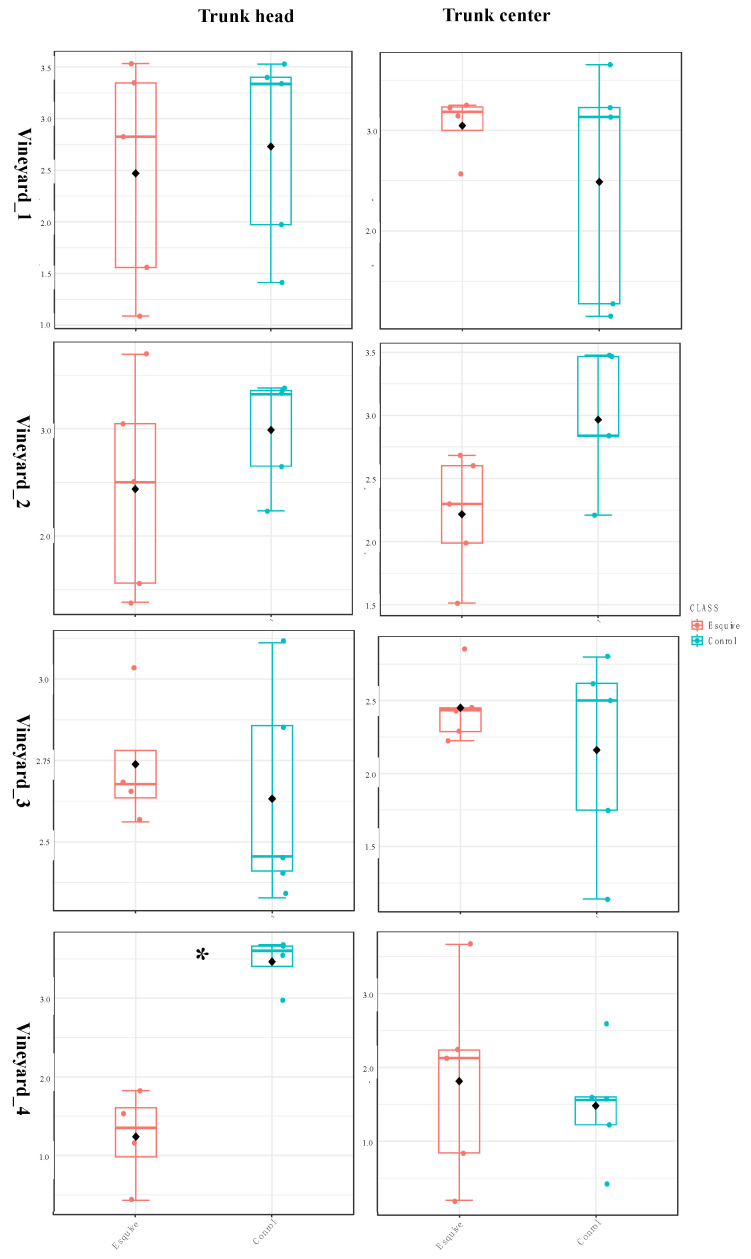
Boxplot illustrating the differences in Shannon diversity measures of the bacterial communities in the grapevine healthy wood of Esquive^®^ WP-treated and control plants at 2 months post-inoculation (T1). * Indicates significant differences between treatments (*p* < 0.01).

In all vineyards, the results of the beta and alpha diversity analyses showed that the bacterial and fungal communities did not significantly differ in the wood of treated plants and control ones at 10 months post-inoculation (T2), irrespective of the studied tissues (Table S2). However, at 2 months post-inoculation (T1), a few differences in microbial communities were observed in two vineyards (Vineyard_3 and Vineyard_4) out of the four studied vineyards. The comparison of the alpha diversity indexes (Shannon and observed) between wood samples treated or not with Esquive^®^ WP showed that the biocontrol product did not significantly affect the fungal communities in Vineyard_1, Vineyard_2, and Vineyard_4. However, in Vineyard_3, the Shannon index decreased significantly at the trunk head level in samples treated with Esquive^®^ WP compared to control ones (*p* = 0.001). (Figure 6 and Table S2A). Regarding the bacterial communities, a similar result was observed. Indeed, in Vineyard_4, at the trunk head level, the Shannon index was significantly lower in the treated grapevine than the control ones at T1 (*p* = 0.001) (Figure 7 and Table S2B). However, no significant differences were observed in the wood bacterial microbiome between the Esquive^®^ WP-treated and control plants in Vineyard_1, Vineyard_2, and Vineyard_3, irrespective of the studied tissue. Concerning the beta diversity of the fungal and bacterial communities, no significant effect of the treatment was observed (Table S3).

#### 3.6.2. Esquive^®^ WP Affects Temporary Wood Microbial Abundance Two Months Post Treatments

**Figure 8:**
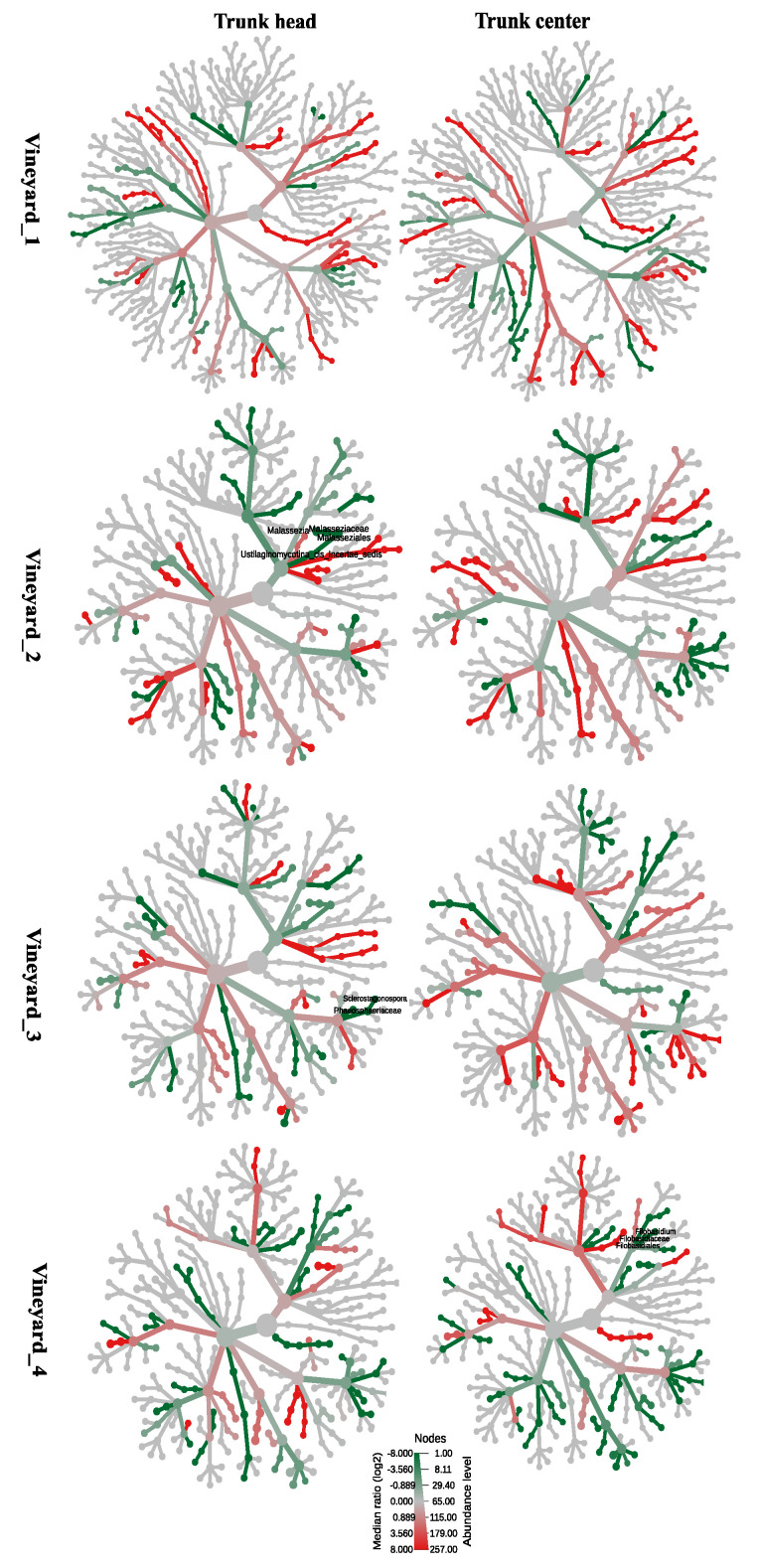
Heat trees of factor treatment. Heat trees report the effect of treatment on a hierarchical structure of taxonomic fungal classifications in Esquive^®^ WP-treated and control plants 2 months post-inoculation. Each cladogram shows pairwise comparisons between treatments (Esquive^®^ WP vs. control) in the different vineyards on two different tissues (trunk head and trunk center). The indicated taxa with red nodes were significantly abundant in the Esquive^®^ WP-treated plants, while green nodes were significantly more abundant in fungal communities of control plants.

**Figure 9:**
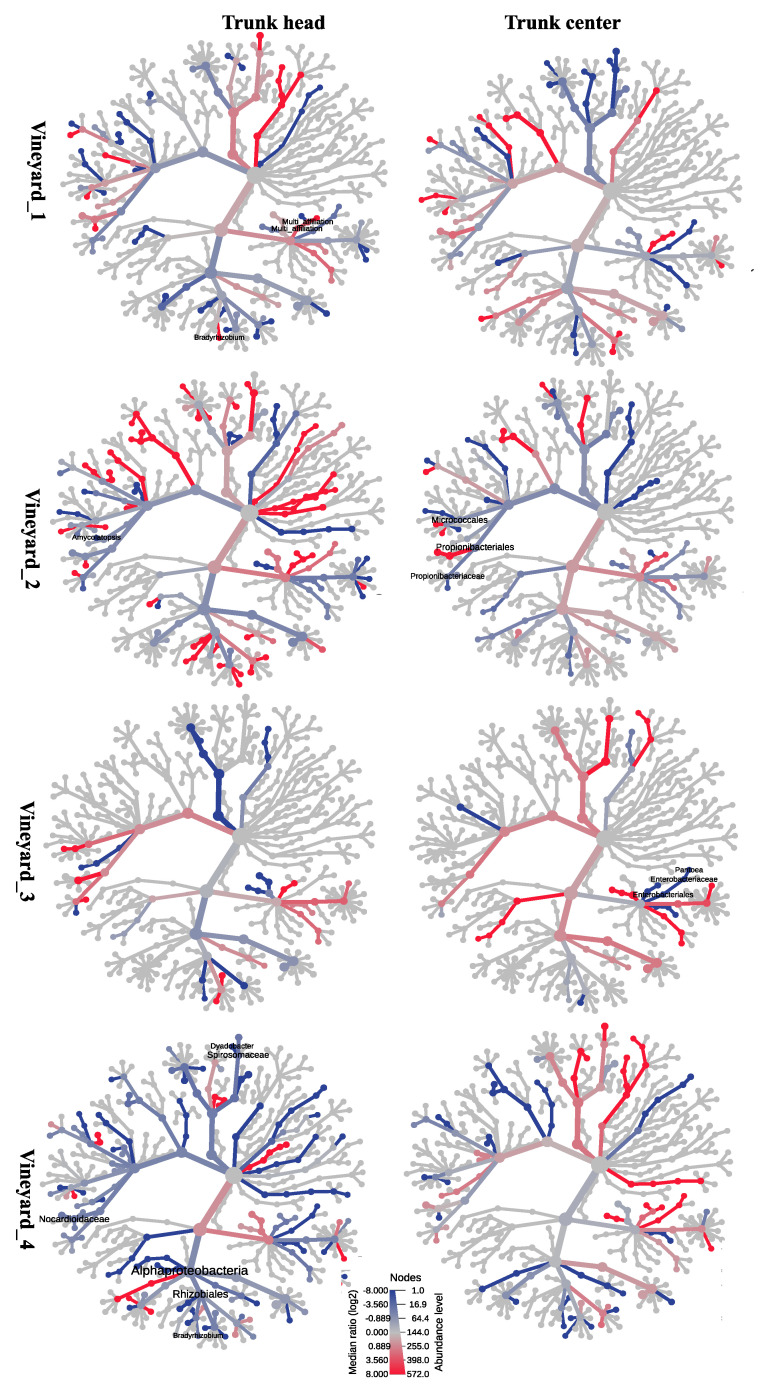
Heat trees of factor treatment. Heat trees report the effect of treatment on a hierarchical structure of taxonomic bacterial classifications in Esquive^®^ WP-treated and control plants 2 months post-inoculation. Each cladogram shows pairwise comparisons between treatments (Esquive^®^ WP vs. control) in the different vineyards on two different tissues (trunk head and trunk center). The indicated taxa with red nodes were significantly abundant in the Esquive^®^ WP -treated plants, while green nodes were significantly more abundant in bacterial communities of control plants.

In order to assess if the Esquive^®^ WP treatment impacted grapevine wood microbiome, heat trees comparing the taxonomic abundance of taxa from each of the conditions (treatment and control) were carried out in the four vineyards at the trunk head and trunk center levels, 2 (Figure 8 and Figure 9) (T1) and 10 months (T2) (Figures S4 and S5) post-inoculation. The heat trees were filtered only to show results with significant differences (Wilcoxon test, *p* < 0.01). As shown above for the microbial diversity (Table S2), in all vineyards, Esquive^®^ WP application on grapevine pruning wounds did not significantly affect the abundance of fungal and bacterial communities at T2 (Figures S4 and S5), irrespective of the studied tissue. However, 2 months following treatment, a few significant differences in microbial communities were observed. For fungal communities, significant differences were observed only in young vineyards (Vineyard_2, Vineyard_3, and Vineyard_4) (Figure 8). As a matter of fact, the taxonomic hierarchical data comparison of the fungal communities in Esquive^®^ WP-treated plants revealed a significantly lesser abundance (green lines) of *Malassezia* under the family Malasseziaceae (within the order Ustilaginomycotina), *Sclerostagonospora* under the family Phaeosphaeriaceae, and *Filobasidium* under the family Filobasidiaceae (within the order Filobasidiales) than in the untreated control (Figure 8) in Vineyard_2, Vineyard_3, and Vineyard_4, respectively. These significant effects of the biocontrol product on the abundance of certain fungal genera were observed either at the trunk head level (Vineyard_2, Vineyard_3) or the trunk center level (Vineyard_4) but not in both. Regarding the abundance of the *Trichoderma* genus in grapevine healthy wood, unexpected results were observed. In all studied vineyards, no significant differences in *Trichoderma* relative abundance were observed in plants treated with Esquive^®^ WP compared to the controls in both tissues, irrespective of the sampling time point.

The treatment effect on the bacterial community is represented in the heat trees (Figure 9). The results pointed out that significant differences in certain bacterial taxa abundance were observed in all the studied vineyards. In particular, Figure 9 displays Vineyard_1, the increase (red lines) of *Bradyrhizobium* taxa in Esquive^®^-treated plants with respect to the control plants at the cordon level. In contrast, bacterial communities in the Esquive^®^-treated plants had a significantly lower abundance (blue lines) of *Amycolatopsis* and *Pantoea* under the family Enterobacteriaceae within the bacterial order Enterobacteriales than the untreated controls in Vineyard_2 and Vineyard_3, respectively. Moreover, in Vineyard_4, the abundance of *Bradyrhizobium* within the order Rhizobiales and *Dyadobacter* under the family Spirosomaceae within the order Cytophagales decreased in Esquive^®^-treated plants compared to the controls (Figure 9). As for the fungal communities, these significant effects of the biocontrol product on the abundance of certain bacterial genera were observed either at the trunk head level (Vineyard_1, Vineyard_2, and Vineyard_4) or the trunk center level (Vineyard_3) (Figure 9).

## 4. Discussion

Biocontrol products are increasingly applied in vineyards worldwide in an attempt to reduce infection by pathogens associated with GTDs [15,17]. Esquive^®^ WP, based on the *T. atroviride* strain I1237, is the first registered product used to control pathogens involved in GTDs in France. The effectiveness of this product in limiting the incidence of pathogens involved in GTDs has already been well studied [25,26,27,28,29,49].

Aside from this, in recent years, grapevine wood microbiome profiling has become of increasing interest due to its involvement in GTDs [50,51,52,53,54,55,56,57] and its contribution to grapevine health [58,59]. However, little is known about the effect of biocontrol product application on the non-targeted wood microbiome, especially over a long-term period of years.

In this study, we aimed to analyze the impact of Esquive^®^ WP applications on the indigenous grapevine wood microbiota after a 6-year period of treatment. Understanding this aspect is crucial to evaluate the environmental risk of the biocontrol product. To the best of our knowledge, this is the first experiment that studies the effect of a biocontrol agent on grapevine microbiome after such a long period of treatment (a 6-year period of treatments).

To achieve this objective, the effect of the Esquive^®^ WP treatment on bacterial and fungal communities of grapevine inner healthy wood was evaluated in grapevine from three different French cultivars located in geographically distinct viticultural zones. The Chardonnay cultivar was selected in both Vineyard_1 and Vineyard_2, and Cabernet Sauvignon and Melon de Bourgogne were studied in Vineyard_3 and Vineyard_4, respectively. The first result of our study was that the Esquive^®^ WP pruning wound treatment had no significant effect on inner wood necroses, which depend mainly on the grapevine’s age and cultivar. For the same cultivar (i.e., Chardonnay), the wood necroses observed in 24-year-old plants were significantly higher than those measured in 14-year-old plants. Plant age is obviously a critical factor for GTD development. In order to explain the grapevine age implication in GTDs, Mugnai et al. [60] detailed that the higher incidence of esca in old vineyards can be due to the fact that younger affected plants do not yet show the whole range of internal and external esca symptoms. Regarding plants of the same age but of different cultivars, the wood necrosis area measured in the Melon de Bourgogne cultivar (Vineyard_4) was significantly higher than those measured in Chardonnay (Vineyard_1 and Vineyard_2) and Cabernet Sauvignon (Vineyard_3) cultivars. In addition, significant differences in the wood necrosis length were observed in plants of the same age and different cultivars. This result is consistent with those obtained in previous studies, indicating that the length of inner wood necrosis caused by pathogens involved in GTDs is cultivar-dependent [61,62,63]. Recently, Etienne et al. [64] reported that the esca incidence is higher in the Melon de Bourgogne cultivar than in Cabernet Sauvignon and Chardonnay ones.

Regarding the *Trichoderma* species, the results showed that their abundance level is low in our samples. This result is consistent with previous studies, based on DNA metabarcoding, showing that the relative abundance of *Trichoderma* taxon in grapevine wood microbiome is very low [51,54,65,66]. Surprisingly, even after the biocontrol product application, *T. atroviride I*-1237 was not detected by qPCR—or was only at very low levels in the inner tissues of Esquive-treated plants. Moreover, the microbiome analysis showed that the *Trichoderma* genus was not presented among the most abundant taxa in the treated plants. In order to explain this result, it could be hypothesized that the fungus preferably colonized the pruning wounds on which it was applied. However, in this study, sampling was carried out in the inner wood at two trunk levels (trunk head and trunk center) (Figure S1).

Regarding the grapevine healthy wood microbiome analysis, based on the use of Miseq next-generation sequencing, the first relevant result we obtained was that the fungal and bacterial microbiome were mainly affected by vineyard factors (which include both the cultivar and geographical location). When the vineyards were compared, the significant differences in bacterial and fungal diversities indicated a major effect of sampling location/cultivar. This agrees with previous studies on grapevine microbiome showing that the sampling location and/or cultivar are known to be strong predictors of microbial diversity in multiple grapevine organs [51,54,67,68,69,70]. However, in addition to different geographical locations and cultivars, certain other factors may have contributed to the observed variability, such as the grapevine’s age. In our study, the beta diversities of fungal communities in young (14-year-old) and old (24-year-old) Chardonnay plants were significantly different. This result has not been observed in bacterial communities. Our observation is not in accordance with those obtained by Kraus et al. [71], who reported that a culturable endophytic fungal community in healthy grapevines is not affected by a grapevine’s age.

Regarding the microbiome wood profile, different bacterial genera such as *Pseudomonas*, *Massilia*, *Sphingomonas*, *Friedmaniella,* and *Pantoea* taxa were found in high relative abundance. These different taxa have already been identified when investigating the grapevine’s wood microbiome [53,57,65,72,73]. The relative abundance of these taxa in grapevine wood is different, according to studies. In our study, while *Pseudomonas* is the most abundant bacterial genus in three of the four studied vineyards, the *Massilia* genus was the most abundant only in one. In line with our findings, Niem et al. [65] showed that *Pseudomonas* dominated the bacterial community in healthy grapevine tissues in two different Australian vineyards, comprising 56–74% of the total bacterial population. Recently, Adejoro et al. [57] confirmed that the *Pseudomonas* and *Massilia* genera are among the most abundant bacterial taxa in the wood of GTD symptom-free grapevines. However, *Pantoea* and *Sphingomonas* were the two most abundant taxa in the non-necrotic wood of healthy and diseased grapevines at the cordon level [53]. Concerning fungal taxa, the abundance profiles of healthy grapevine wood in all vineyards showed a predominance of fungal GTDs, such as *Diplodia*, *Neofusicoccum,* and *Phaeomoniella*. Our result is in accordance with previous studies showing that GTD pathogens were abundant in the woody tissue of both symptomatic and asymptomatic grapevines from different vineyards [28,49,50,53,54,65,72,73,74,75,76]. Among the GTD pathogens, *Phaeomoniella* was considered the predominant taxa in grapevine wood of symptomatic and asymptomatic grapevines [51,53,65]. This observation has not been confirmed here, as the abundance level of Botryosphaeriaceae (*Neofusicoccum* and *Diplodia*) fungi was higher than *Phaeomoniella* in the wood samples of asymptomatic grapevines. These differences in relative abundance might be explained by the origin of the wood samples. As an example, [75] showed that *P. chlamydospora* was far more abundant in border tissue (between white rot and non-necrotic tissue) than in non-necrotic wood samples.

Interstingly, the 6-year grapevine pruning wound treatment with the biocontrol product did not significantly affect the biodiversity of the healthy wood microbiome, confirming that the geographical sampling site/cultivar has a key impact on the microbial diversity [67]. A similar conclusion was achieved by Perazzoli et al. [77], which assumed that the phyllosphere microbiota of the grapevine was little affected by the treatments. Phyllosphere microbiota mainly differed depending on the geographical location of the grapevine.

In our study, following the Esquive^®^ WP applications, temporary changes in the plant microbiome were observed. In the Gironde and Loire-Atlantique vineyards, a significant modification of OTU richness and evenness was observed between treated and non-treated grapevines at 2 months after Esquive^®^ WP treatment and not after 10 months of biocontrol product application. The same result was observed when the abundance of fungal and bacterial taxa was studied. In fact, the application of the biocontrol product negatively affected the abundance of certain fungi (*Malassezia*, *Sclerostagonospora,* and *Filobasidium*) and bacteria (*Bradyrhizobium*, *Amycolatopsis*, *Pantoea*, and *Dyadobacter)* genera. On the contrary, in Vineyard_1, the application of Esquive^®^ WP increased the abundance of the bacterial genus *Bradyrhizobium*. Consequently, the significant modification in the richness and abundance of microbial communities due to Esquive^®^ WP treatment is clearly transient. This demonstrates the natural capacity of fungal and bacterial communities to become resilient and return to a balanced state. After the Esquive^®^ WP application, these modified taxa are not known to be involved in grapevine trunk diseases or as biocontrol agents. Similar observations have also been reported that the introduction of *T. atroviride* SC1 had an effect on the grapevine’s soil microbiome during the first two weeks following inoculation. However, at later dates, environmental conditions had a higher influence on the surveyed communities than the BCA application [78].

Recently, numerous studies have assessed the impact of the BCA application on grapevine microbiome [50,51,79,80]. The results obtained were different, depending on many factors, such as the BCA, the age of the grapevine, the studied tissue, the time of sampling, and the microbial communities targeted (bacteria or fungi). Leal et al. [80] showed that the *T. atroviride* strain SC1 application had negative impacts on fungal diversity, but it did not affect bacterial diversity. For example, less abundance of some biological control fungal species, such as *Fusarium* and *Clonostachys*, were observed in the treated soil. In the same study, the inoculation of *Bacillus subtilis* PTA-271 did not affect the grapevine’s rhizosphere microbiome. Del Frari et al. [51]. Showed that pruning wound-protection products, including Esquive^®^ WP, induced alterations in the fungal communities of 1-year-old grapevine canes 3 months after inoculation. As an example, *Epicoccum* sp., a fungal genus with biological control potential, was less negatively affected by Esquive treatment. Recently, Pulcini et al. [79] showed that the application of a commercial biostimulant on the grapes did not have any significant impact on this microbiota after a 2-year treatment period.

## 5. Conclusions

In conclusion, our results showed that recurrent applications (6 years of consecutive annual treatment) of the biocontrol product Esquive^®^ WP do not lead to significant disturbances in the microbial communities of healthy vinewood. Only a transient change in microbial communities was observed after the Esquive^®^ WP treatments before its return to a balanced state. This main result confirms that the resilience of a well-established microflora allows it to stand the introduction of a biocontrol agent. As such, we showed an essential criterion for a BCA, namely its ability to protect grapevines against pathogens involved in GTDs without inducing significant and persistent changes in native microbial populations.

## Figures and Tables

**Table 1:** PERMANOVAs of beta diversity indexes for fungal and bacterial communities: Treatment (Esquive^®^ WP and control), sampling time (2 and 10 months after inoculation), tissue (trunk head and trunk center), and vineyards (Vineyard_1, Vineyard_2, Vineyard_3, and Vineyard_4). Significant *p*-values are shown in bold.

	Fungi		Bacteria	
	R^2^	*p*-Value	R^2^	*p*-Value
Treatment	0.007	0.281	0.006	0.476
Sampling Time	0.011	0.045	0.044	**<0.001**
Tissue	0.022	**0.001**	0.014	**<0.01**
Vineyard	0.22	**0.001**	0.154	**<0.001**

**Table 2:** Experimental factors predicting alpha-diversity of wood associated fungal and bacterial communities. Treatment (Esquive^®^ WP and control), sampling time (2 and 10 months after inoculation), tissue (trunk head and trunk center), vineyards (Vineyard_1, Vineyard_2, Vineyard_3, and Vineyard_4). Significant *p*-values are shown in bold.

	Fungi		Bacteria	
	Observed	Shannon	Observed	Shannon
Treatment	0.12	0.26	0.45	0.31
Sampling Time	0.8	0.27	**<0.001**	0.068
Tissue	**0.004**	0.58	**<0.001**	**<0.01**
Vineyard	0.15	**<0.001**	**<0.001**	**<0.01**

## Data Availability

The original contributions presented in this study are included in the article/Supplementary Material. Further inquiries can be directed to the corresponding author.

## Supplementary Materials

The following supporting information can be downloaded at: https://www.mdpi.com/article/10.3390/jof10080566/s1, Figure S1: Wood sampling from the inner trunk at two different positions (Trunk head and Trunk center); Figure S2: A longitudinal trunk section showing an example of the measured wood necrosis; Figure S3: Boxplot illustrating the differences in alpha diversity measures (Observed index) of the fungal (A) and bacterial (B) communities between vineyards; Table S1: Pairwise PERMANOVAs of beta diversity indexes between vineyards for fungal and bacterial communities; Table S2: Alpha richness and diversity indexes for fungal (A) and bacterial communities (B) of treatment effect; Table S3: PERMANOVAs of beta diversity indexes for fungal and bacterial communities; Figure S4: Heat tree of factor Treatment; Figure S5: Heat tree of factor Treatment.
