# Supplementary material for: Impact of the Biocontrol Product, Esquive® WP, on the Indigenous Grapevine Wood Microbiome after a 6-Year Application Period
Source: J Fungi (Basel). 2024 Aug 11;10(8):566. doi: 10.3390/jof10080566 (PMC11355725; doi:10.3390/jof10080566)
Supplement: Supplementary file 1 [file jof-10-00566-s001.zip › jof-3073789-supplementary.pdf]

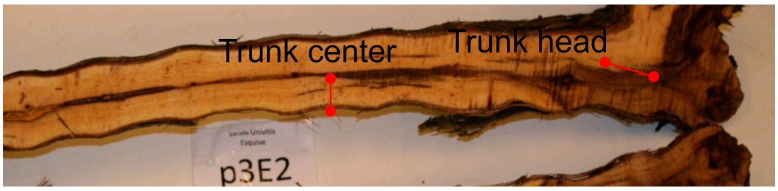

Figure S1: Wood sampling from the inner trunk at two different positions (Trunk head and Trunk center)

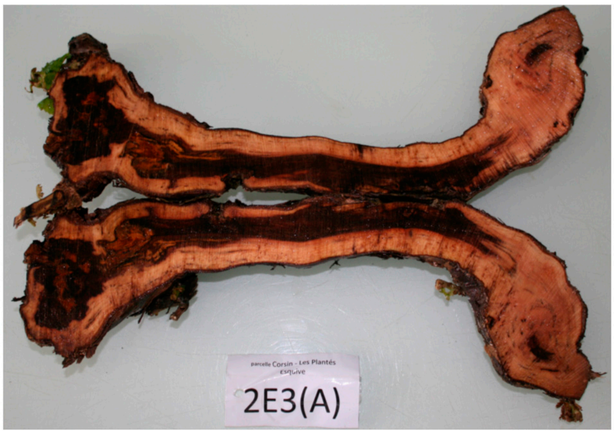

Figure S2: A longitudinal trunk section showing an example of the measured wood necrosis

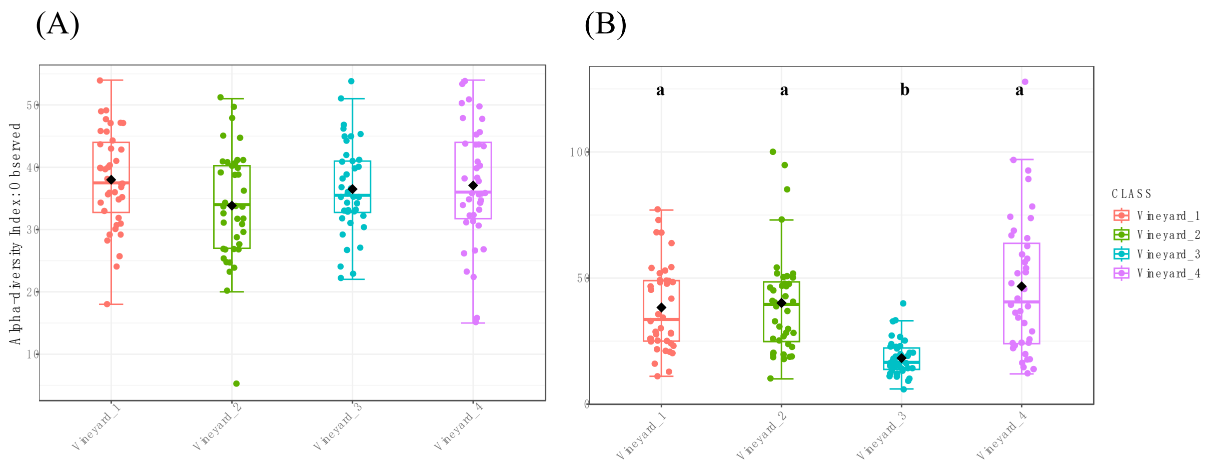

Figure S3: Boxplot illustrating the differences in alpha diversity measures (Observed index) of the fungal (A) and bacterial (B) communities between vineyards

Table S1: Pairwise PERMANOVAs of beta diversity indexes between vineyards for fungal and bacterial communities.

	Fungi	Bacteria
	**p-value**	**p-value**
**Vineyard_1 *vs* Vineyard_2**	0.001	0.07
**Vineyard_1 *vs* Vineyard_3**	0.001	0.001
**Vineyard_1 *vs* Vineyard_4**	0.001	0.001
**Vineyard_2 *vs* Vineyard_3**	0.001	0.001
**Vineyard_2 *vs* Vineyard_4**	0.001	0.001
**Vineyard_3 *vs* Vineyard_4**	0.001	0.001

Table S2: Alpha richness and diversity indexes for fungal (A) and bacterial communities (B) of treatment effect. T1 and T2 correspond to sampling time points, 2- and 10-months after Esquive® WP treatment, respectively. Significant *p values* are shown in bold.

(A)

	Bacteria							
	Observed				Shannon			
	T1		T2		T1		T2	
	Trunk head	Trunk center	Trunk head	Trunk center	Trunk head	Trunk center	Trunk head	Trunk head
**Vineyard_1**	0.82	0.96	0.46	0.2	0.7	0.36	0.72	0.26
**Vineyard_2**	0.32	0.81	0.67	0.6	0.3	0.04	0.84	0.45
**Vineyard_3**	0.63	0.7	0.97	0.12	0.58	0.42	0.27	0.11
**Vineyard_4**	0.97	0.35	0.36	0.23	**0.001**	0.64	0.52	0.83

(B)

	Fungi							
	Observed				Shannon			
	T1		T2		T1		T2	
	Trunk head	Trunk center	Trunk head	Trunk center	Trunk head	Trunk center	Trunk head	Trunk head
**Vineyard_1**	0.9	0.34	0.79	0.55	0.61	0.6	0.2	0.2
**Vineyard_2**	0.99	0.77	0.6	0.67	0.49	0.71	0.95	0.72
**Vineyard_3**	0.85	0.39	0.59	0.7	**0.01**	0.54	0.83	0.75
**Vineyard_4**	0.1	0.1	0.4	0.16	0.67	0.21	0.9	0.9

Table S3: PERMANOVAs of beta diversity indexes for fungal and bacterial communities. T1 and T2 correspond to sampling time points, 2- and 10-months after Esquive® WP treatment, respectively. Showed values correspond to *p values*.

	Bacteria				Fungi			
	T1		T2		T1		T2	
	Trunk head	Trunk center	Trunk head	Trunk center	Trunk head	Trunk center	Trunk head	Trunk head
**Vineyard_1**	0.42	0.65	0.79	0.45	0.5	0.39	0.68	0.81
**Vineyard_2**	0.132	0.125	0.878	0.41	0.11	0.4	0.28	0.35
**Vineyard_3**	0.26	0.07	0.06	0.57	0.6	0.39	0.8	0.71
**Vineyard_4**	0.06	0.76	0.73	0.46	0.833	0.82	0.8	0.35

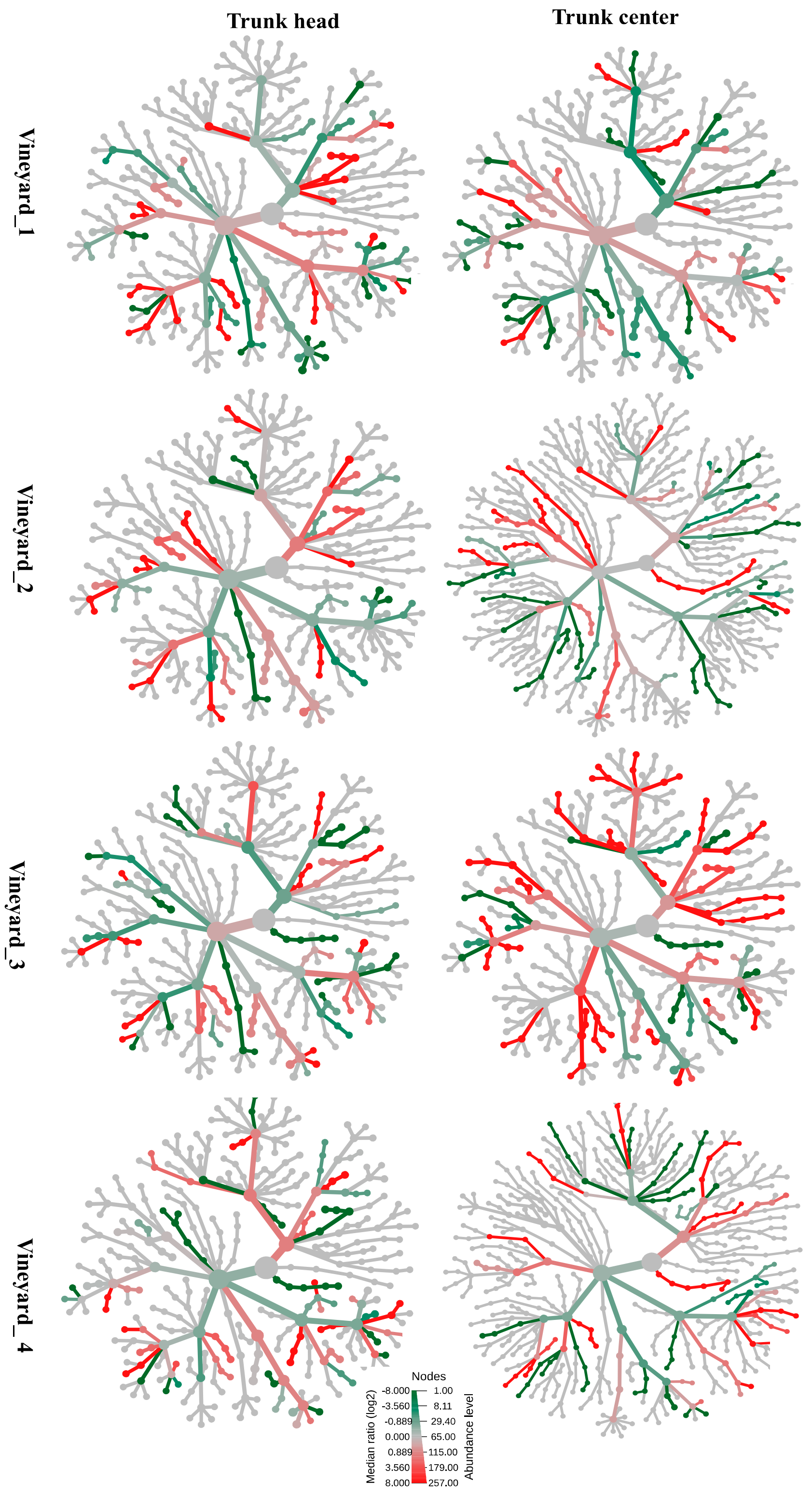

Figure S4: Heat tree of factor Treatment. Heat Trees report the effect of treatment on hierarchical structure of taxonomic fungal classifications in Esquive® WP treated and control plants, 10 months post-inoculation. Each cladogram shows pairwise comparisons between treatments (Esquive® WP vs control), in the different vineyards, at two different tissues (trunk head and trunk center). The indicated taxa with red nodes were significantly abundant in the

Esquive® WP -treated plants, while green nodes significantly more abundant in fungal communities of control plants.

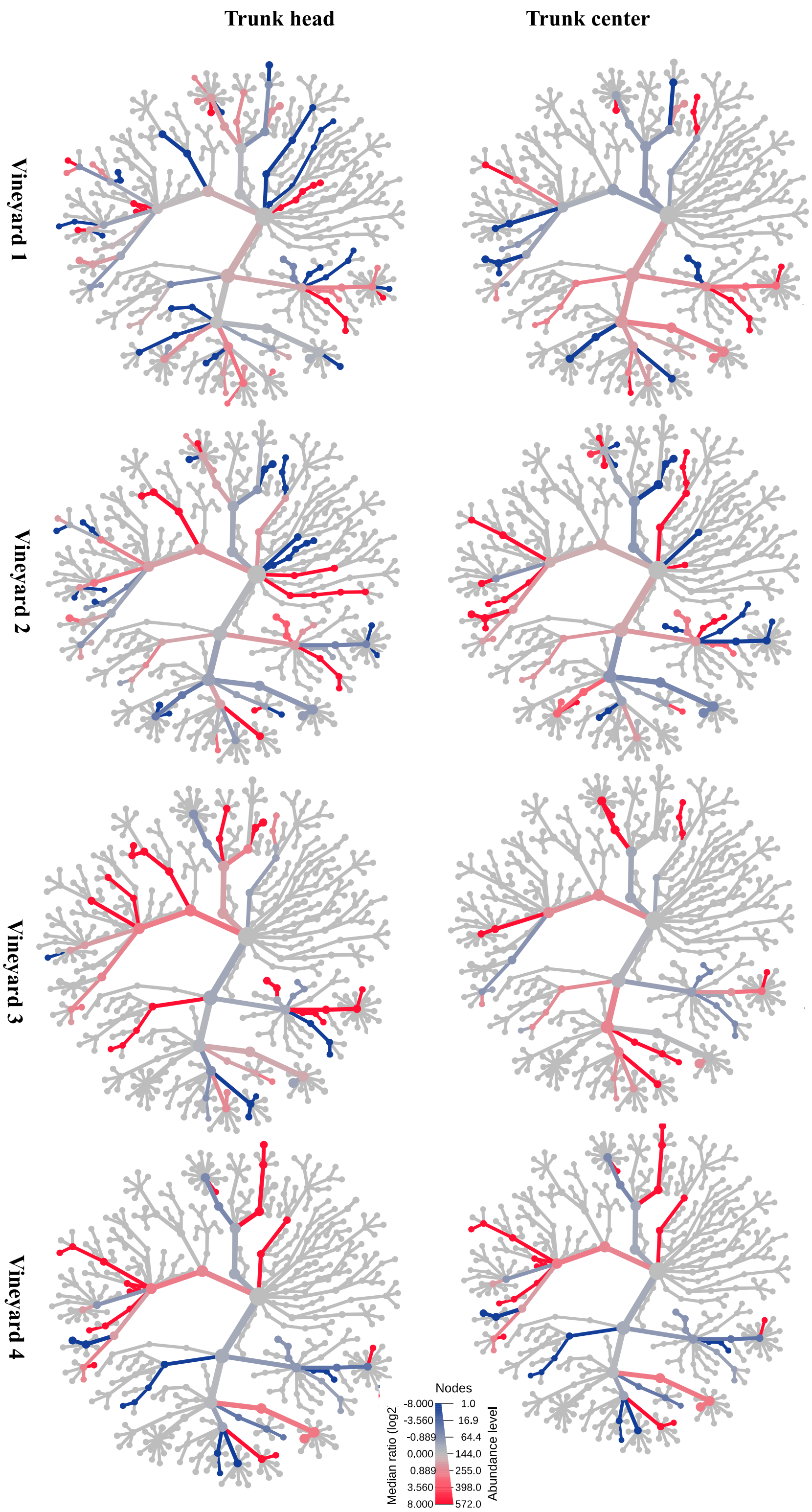

Figure S5: Heat tree of factor Treatment. Heat Trees report the effect of treatment on hierarchical structure of taxonomic bacterial classifications in Esquive® WP treated and control plants, 10 months post-inoculation. Each cladogram shows pairwise comparisons between treatments (Esquive® WP vs control), in the different vineyards, at two different tissues (trunk head and trunk center). The indicated taxa with red nodes were significantly abundant in the

Esquive® WP -treated plants, while green nodes significantly more abundant in bacterial communities of control plants.
